# Follow-up care of rare diseases in odontology: a public health issue

**DOI:** 10.1186/1746-160X-8-S1-P11

**Published:** 2012-05-25

**Authors:** S Toupenay, M de la Dure, N Razanamihaja, A Berdal, M-L Boy-Lefèvre

**Affiliations:** 1 Department of Public Health, Dental Faculty, University of Paris Diderot, France; 2 Reference Centre of Rare Diseases of Face and Buccal Cavity, Paris Hospitals, France; 3 INSERM Cordeliers Biomedical Research Centre, Paris, France

## 

This study aims at providing new data relating to rare diseases with or without bucco-dental component. A national investigation based on a questionnaire has been carried out in France and a clinical study has been completed with the patients consulting the national centre of rare malformations of the face and the buccal cavity, in particular oligodontia.

The results revealed a difficult and complex course of care for all the patients suffering from rare diseases. The oral health quality is not linked to the severity of the bucco-dental component. An unfavorable medico-economic impact has been noted in the treatment of numeric anomalies of teeth, including oligodontia. Proposals have been raised to improve the follow-up care of these patients on the basis of the current evolution of health policy.

